# Promoting mental health in the police sector: an integrated model of resilience, organisational support and emotional literacy

**DOI:** 10.3389/fpsyg.2026.1771519

**Published:** 2026-02-13

**Authors:** Vanesa Berlanga Silvente, Santiago Gascón-Santos, Yago Pérez-Montesinos

**Affiliations:** 1 Departamento de Educación y Humanidades, Universidad Abat Oliba CEU, Barcelona, Spain; 2 Departamento de Psicología y Sociología, Universidad de Zaragoza, Zaragoza, Spain

**Keywords:** emotional literacy, mental health, operational stress, organisational support, police, police wellbeing, resilience, work environment

## Abstract

Police work takes place in highly demanding contexts, characterised by frequent exposure to critical events, time pressure, physical risk and high emotional demands. These factors increase the risk of chronic stress, operational fatigue and professional burnout. Despite growing interest in police wellbeing, there is still a lack of an integrated framework that articulates the main protective factors and guides preventive policies. This article aims to synthesise recent literature on mental health in police forces and propose an operational model for promoting wellbeing tailored to the needs of this group. A narrative review of the literature was conducted through a selective search in PubMed, PsycINFO, Scopus, and Dialnet (2010–2025). Studies focusing on police resilience, organisational support, emotional competencies, operational stress, leadership, and organisational climate were included. The studies analysed identify five key protective factors for police mental health: (1) individual and collective resilience; (2) social and organisational support; (3) emotional literacy and affective regulation; (4) sense of purpose and professional identity; and (5) psychosocial climate and healthy leadership. Based on these axes, the Integrated Model of Police Mental Health (IPMHM) is proposed, which articulates interventions at the individual, group and organisational levels. The results suggest that promoting mental health in police forces requires systemic approaches that integrate personal competencies with safe organisational environments and professional cultures oriented towards well-being, highlighting the role of leadership, continuous training and peer support.

## 1 Introduction

Police work takes place in a uniquely complex environment where operational, emotional and ethical demands converge. Unlike other professions in the public sector, police work places officers in situations where they must balance protecting citizens, managing conflicts and making immediate decisions under pressure (Bishopp et al., 2020). This combination makes the police a group that is particularly exposed to prolonged psychological stress. Numerous studies have pointed out that continuous exposure to risky situations, aggressive or unregulated behaviour by third parties, and scenes of human suffering increases the emotional burden on police personnel and can affect their psychological well-being (Hartley et al., 2012; Garbarino et al., 2013; Violanti et al., 2017).

In addition to the risks associated with critical incidents—such as assaults, pursuits, or accidents—police work also involves activities that, although less dramatic, cause sustained wear and tear: long hours, shift work, frequent contact with people in crisis, accumulated bureaucracy, and the need to maintain constant attention. Over time, this sustained pressure can lead to emotional fatigue, irritability, sleep difficulties, feelings of detachment and, in the most extreme cases, burnout or trauma-related symptoms (McCanlies et al., 2018; Gascon-Santos et al., 2024). However, it is important to emphasise that the presence of these conditions does not make police officers an intrinsically vulnerable group; what the literature reveals is that the psychological impact depends on the interaction between the individual and their professional context.

Beyond policing, research conducted in other public service professions exposed to aggression has shown similar patterns of psychological impact. For instance, Gascon-Santos et al. (2024) found that repeated exposure to aggressions among primary care health professionals was associated with higher levels of post-traumatic stress symptoms and burnout. These findings.

reinforce the relevance of analysing aggression-related stress as a cross-cutting occupational risk in professions involving direct interaction with the public, including policing.

In this regard, research over the last fifteen years has begun to shift from an approach focused exclusively on risks to a more balanced perspective oriented towards protective factors. This transformation has been driven by accumulated evidence showing that, even in high-pressure contexts, many agents maintain healthy levels of emotional and professional functioning. This capacity for adaptation has led to a more detailed study of elements such as resilience, social and organisational support, emotional literacy and police culture as modulators of well-being (Andersen et al., 2015; Arble and Arnetz, 2017).

Among the most consistent factors, resilience occupies a central place. In the police field, it is not understood as an innate trait, but rather as a dynamic process that combines cognitive skills, coping strategies, emotional flexibility, and team cohesion. Recent research shows that programmes aimed at strengthening resilience not only reduce psychological distress, but also increase operational confidence and a sense of self-efficacy in the face of critical events (Schaible and Gecas, 2010). This suggests that resilience is not only an individual resource, but also a collective one: colleagues, supervision, and leadership directly influence how officers interpret and manage stressful situations.

Added to this component is organisational support, an element that has been extensively studied in occupational psychology. In police forces, perceived support from the institution—especially from immediate superiors—is associated with lower levels of burnout, greater job satisfaction, and a better internal climate (Shane, 2010). Officers who feel that their organisation supports them, recognises their work, and provides safe spaces to express difficulties show better emotional adjustment, even when faced with traumatic incidents. Conversely, a rigid, overly hierarchical or insensitive work culture can increase feelings of isolation and exacerbate the impact of operational stress (Shane, 2010; Manzoni and Eisner, 2006; Tucker, 2015). Another fundamental aspect is emotional literacy, understood as the ability to identify, understand and regulate one’s own and others’ emotions. Although this area was not traditionally part of police training, it has become increasingly important in recent years.

Studies conducted on European and North American police forces show that emotional regulation predicts the ability to de-escalate conflicts, make decisions under pressure, and adapt after complex interventions. Likewise, emotional literacy is associated with a lower risk of impulsive behaviour, excessive reactivity, or empathy fatigue. At the same time, the literature has highlighted the value of a sense of purpose. Police professional identity is often deeply linked to values such as service, protection and justice. When these values are aligned with everyday practice, officers experience a deep sense of meaning in their work, which acts as a buffer against stress (Bakker et al., 2005; Schaufeli et al., 2006; Kuo, 2015). Conversely, when there is a perceived disconnect between the institutional mission and the actual working conditions, frustration, cynicism or loss of motivation may arise (Bakker et al., 2005).

Finally, the psychosocial climate and leadership play a decisive role. A clear, predictable, fair and cohesive working environment promotes well-being. Middle managers, in particular, are key figures: their communication style, their ability to recognise effort and their sensitivity to the emotional burden of the team directly influence the mental health of staff (Hartley et al., 2012; Carleton et al., 2018). In fact, emerging studies indicate that transformational leadership — based on trust, support and shared vision — reduces operational stress and improves the atmosphere of unity (Aymerich et al., 2021).

Despite these findings, the literature remains fragmented and scattered across different approaches. A conceptual framework is needed that clearly synthesises the protective factors identified and integrates them into an operational model that is accessible to police institutions. This article responds to this need by proposing a model that articulates five pillars—resilience, organisational support, emotional literacy, sense of purpose, and work climate—with the aim of guiding preventive programmes, continuing education, and wellness strategies specifically designed for police forces.

This proposal is aligned with the organisational perspective on burnout proposed by Leiter and Maslach (2004, 2016), according to which psychosocial risks are not only a problem for the individual, but mainly for organisational structures, also incorporating an integrative vision based on the interaction between personal and contextual factors (Gascon-Santos et al., 2024).

## 2 Method

This research is based on a narrative review of the literature, an appropriate approach when the objective is not to quantify effects or compare specific interventions, but rather to understand, integrate, and synthesise existing knowledge about a complex phenomenon such as mental health in the police environment. This type of review allows for the incorporation of diverse perspectives, drawn from occupational psychology, policing studies, organisational leadership, and the neuroscience of stress, offering a broader view than that provided by a strictly systematic design (Leiter and Maslach, 2004, 2016).

### 2.1 Search strategy

To ensure consistency and rigour in the review, a structured search was conducted in four main databases: PubMed, PsycINFO, Scopus, and Dialnet. These platforms offer a balanced combination of biomedical, psychological, and criminological literature, as well as Spanish scientific output. The search covered the period 2010–2025, allowing for the inclusion of both seminal works from the last decade and more recent contributions related to police resilience, emotional management, and the psychosocial impact of operational work.

Combinations of terms reflecting the study’s areas of analysis were used, such as: *police mental health, law enforcement resilience, organisational support for police, critical incident stress, police emotional regulation, police burnout, and police wellness programmes.* In the case of Dialnet, Spanish equivalents were added, such as police mental health, police operational stress, and police resilience.

The selection of keywords was adjusted iteratively as the preliminary reading progressed, allowing for the incorporation of emerging concepts such as emotional literacy and healthy leadership.

### 2.2 Inclusion criteria

To ensure the relevance of the material analysed, selection criteria were established that focused exclusively on the police sector. The following criteria were included:

1. Empirical articles, both quantitative and qualitative, addressing the psychological well- being of local, regional, or national police officers.
2. Narrative or systematic reviews, provided they contribute relevant theoretical syntheses on police mental health.
3. Conceptual studies related to resilience, emotional regulation, work environment, police leadership, or organisational support.
4. Institutional reports issued by organisations such as the International Association of Chiefs of Police (IACP), the National Institute of Justice (NIJ), the FBI Law Enforcement Bulletin (FBI-LEB) or the European Agency for Safety and Health at Work (EU-OSHA), given their influence on professional practices.

Only studies with a focus on promotion or prevention were included; that is, those that analyse protective factors or psychological strengthening strategies, rather than focusing exclusively on pathology.

### 2.3 Exclusion criteria

Studies were excluded that:

- focused on other professional groups (health workers, teachers, military personnel, firefighters),
- addressed only clinical disorders unrelated to police work,
- did not offer information applicable to the operational context of law enforcement agencies,
- or consisted of anecdotal reports or studies without peer review.

This filtering allowed us to focus our analysis on literature directly relevant to the development of the proposed model.

### 2.4 Screening and selection process

Once the articles had been identified, a two-stage screening process was carried out. In the first stage, titles and abstracts were reviewed to exclude irrelevant studies. In the second phase, the selected articles were read in full, and information on objectives, methodology, variables studied, and main conclusions was recorded. This reading allowed for the detection of recurring thematic patterns, as well as differences between studies from European, North American, and Latin American police environments.

The identification of studies from European, North American and Latin American contexts was based on the country of origin of the police forces or institutions described in each article, as explicitly stated in the study samples or institutional settings. This geographical reference was used descriptively to contextualise findings, not as a basis for formal comparative analysis, which remained thematic in nature.

The complete process of identifying, screening, and selecting the studies is summarised descriptively in Table 1. Exclusion decisions at each stage were guided by thematic relevance and operational applicability rather than by formal quantitative thresholds, in line with the narrative review approach.

**Table 1:** Descriptive process of searching, screening, and selecting studies included in the narrative review.

Phase	Description	Records (*n*)
Identification	Records identified in PubMed	72
	Records identified in PsycINFO	54
	Records identified in Scopus	89
	Records identified in Dialnet	31
	Total records identified	**246**
Duplicates	Duplicate records removed	68
	Records after removal of duplicates	**178**
Initial screening	Records excluded after reading the title and abstract (based on relevance and scope criteria)	112
	Articles preselected for full reading	**66**
Full text Reading	Full texts excluded for not meeting inclusion criteria (e.g., pure clinical focus, other professional groups, or low operational applicability)	38
Final inclusion	Studies included in the thematic synthesis	**28**

Source: prepared internally.

At both screening stages, records were excluded based on predefined relevance criteria, including focus on non-police populations, purely clinical perspectives without occupational context, or limited applicability to operational policing. Given the narrative nature of the review, exclusion reasons are reported descriptively rather than quantitatively. Values in bold indicate the final number of articles selected and used in each phase of the review process.

### 2.5 Thematic summary

The information was integrated using inductive thematic synthesis, a common procedure in narrative reviews where findings are grouped around emerging concepts. In this case, the literature showed remarkable convergence around five themes: police resilience, social and organisational support, emotional literacy, sense of purpose, and work climate and leadership. Each of these themes was analysed considering its conceptual definition, the psychological protection mechanisms identified, and its practical applicability in police environments.

The choice of this approach responds to the need to understand police mental health not as a linear phenomenon, but as a network of interrelated factors. This mode of analysis has been recommended in previous studies of occupational well-being in law enforcement agencies (Arble and Arnetz, 2017; Violanti et al., 2017) and provides a solid basis for developing integrated models.

In line with the narrative review approach, no systematic review protocol (e.g., PRISMA) was followed, nor is it intended to provide an exhaustive inventory of all available scientific output, but rather an interpretative synthesis aimed at constructing an applied conceptual model.

### 2.6 Ethical considerations

As this research does not include fieldwork or involve human participants, an assessment by an ethics committee was not necessary. The review is based exclusively on publicly available scientific publications and institutional documents.

## 3 Results

The literature review identified a relatively consistent set of protective factors that recur in studies from different countries and police forces. Although each study approaches police well-being from specific angles, five major themes emerge which, taken together, offer a clear picture of the elements that promote mental health in this group: individual and group resilience, social and organisational support, emotional skills, sense of purpose, and work environment. These axes do not operate in isolation; on the contrary, most studies point to interactions between them, which highlights the need to conceive of police psychological health as a multidimensional phenomenon. A summary of the main studies reviewed and their contribution to the identified thematic areas is presented.

Table 2 presents a selection of the most representative studies included in the narrative review, chosen for their conceptual relevance and illustrative value across the identified thematic areas. The remaining studies (*n* = 10) were also considered in the thematic synthesis but are not displayed in the table to avoid redundancy, in line with the narrative review approach.

**Table 2:** Key studies included in the narrative review and main contributions.

Author(s) and year	Country and sample	Main topic	Key findings
Bakker et al. (2005)	Netherlands – Police officers	Motivation & job resources	Job resources (e.g., meaningful work, recognition) buffer stress and enhance motivation and well-being among police officers.
Schaufeli et al. (2006)	Netherlands – Workers (validation studies; widely applied to police samples)	Work engagement (motivation, meaning, well- being)	Work engagement—defined by vigor, dedication, and absorption—is associated with higher motivation, better psychological well-being, and lower levels of stress and burnout, acting as a protective factor in demanding occupations such as policing.
Arnetz et al. (2009)	USA – Municipal police	Resilience training	Resilience programmes improve coping and reduce post-incident impact.
Shane (2010)	USA – Police officers	Organisational support and stress	Organisational stressors and lack of institutional support are associated with higher stress levels and poorer police performance.
Garbarino et al. (2013)	Italy – National Police	Work-related stress and mental health	A significant relationship exists between workload, stress, and mental health problems.
Papazoglou and Andersen (2014)	Canada / USA	Training in police resilience	Resilience is conceptualised as a skill that can be developed through targeted training programmes.
Ménard and Arter (2014)	USA – Municipal police	Coping and stress	The type of coping strategies influences the incidence of occupational stress.
Manzoni and Eisner (2006)	Switzerland – Police officers	Organisational justice, stress and burnout	Perceived organisational justice and job satisfaction are associated with lower levels of stress, emotional exhaustion and burnout among police officers.
Kuo (2015)	Taiwan – Police officers	Job satisfaction & affective commitment	Job satisfaction is associated with affective commitment, and occupational stressors predict both outcomes.
Tucker (2015)	USA – Police organisations	Perceived organizational support, confidentiality, and stigma (help- seeking)	Higher perceived organizational support and confidentiality are associated with greater willingness to use stress intervention services, while stigma acts as a significant barrier to help- seeking among police officers.
McCanlies et al. (2018)	USA – Police officers	Resilience and social support	Resilience and social support reduce depressive symptoms following exposure to traumatic events.
	USA – Police officers	Emotion regulation and emotional survival	Emotional regulation and emotional survival strategies are associated with better psychological adjustment and reduced stress among police officers.
Stogner and Miller (2020)	USA – Police during COVID- 19	Resilience and mental health	Resilience cushions the psychological impact of extreme working conditions during the COVID-19 pandemic.
Violanti et al. (2017)	USA – Police officers	Reviews on police stressors	Chronic police stressors are identified, highlighting their cumulative impact on physical and psychological health.
Aymerich et al. (2021)	Spain – Catalan police	Transformational leadership training	Training in transformational leadership shows measurable improvements in leadership quality (pre–post design with Subordinate evaluation).
European Agency for Safety and Health at Work (EU-OSHA) (2021)	European Union	Psychosocial risks in policing	Recommends integrating psychosocial risk prevention and promoting a healthy organisational culture within police forces

Source: prepared internally.

The table summarises a selection of the most representative studies found in the search, without being exhaustive, consistent with the narrative review approach.

### 3.1 Individual and collective resilience

The first recurring theme in the studies analysed is the role of resilience. Although the concept has been approached in different ways, a significant portion of the studies reviewed suggest that the ability to adapt to critical situations and maintain stable functioning in the face of adversity is one of the most influential factors in police well-being (Papazoglou and Andersen, 2014; Arble and Arnetz, 2017). In practice, resilience translates into skills such as cognitively reinterpreting stressful events, maintaining perspective in times of high pressure, and resorting to active coping strategies (Habersaat et al., 2015).

Some authors distinguish between individual resilience and collective resilience. The former is linked to personal traits and emotional competencies, while the latter is associated with group cohesion, mutual trust, and a sense of belonging within the unit. Studies conducted on police forces in the United States and Europe have shown that teams with high levels of cohesion experience less emotional exhaustion and deal more effectively with situations of conflict or violence (Violanti et al., 2017; Garbarino et al., 2013).

### 3.2 Social and organisational support

The second thematic axis refers to interpersonal and organisational support, which acts as a key buffer against operational stress. Several studies indicate that support from colleagues, supervisors, and leaders has a direct effect on reducing symptoms of psychological distress, especially after critical incidents (Shane, 2010). Perceived organisational support—the feeling that the institution values and supports the agent—is associated with greater job satisfaction, less burnout, and better emotional functioning. In qualitative studies, agents describe how feeling listened to by their superiors and having safe spaces to express difficulties makes a substantial difference to their daily well-being (Ménard and Arter, 2014; Habersaat et al., 2015).

One relevant line of research is peer support programmes, which have become widespread in Anglo-Saxon countries and are beginning to be implemented in Europe. These programmes enable police officers trained in emotional support to act as a first resource after traumatic interventions, with promising results in reducing acute stress [International Association of Chiefs of Police (IACP), 2019].

### 3.3 Emotional literacy and affective regulation

A third theme that appears repeatedly in the studies reviewed is the importance of emotional skills. Traditionally, police training has focused on tactical and procedural skills, relegating the emotional dimension. However, more recent studies show that the ability to identify, understand and regulate one’s own emotions has a direct impact on conflict resolution, the quality of decision- making and the prevention of impulsive behaviour.

Among the studies reviewed, there is a tendency to suggest that greater emotional literacy reduces defensive or dysregulated responses in high-stress situations and may protect against emotional fatigue and chronic irritability. In the policing literature, emotional regulation skills have been associated with better psychological adjustment and operational functioning in high-demand contexts. In parallel, work engagement—commonly operationalised with the UWES—has been linked to lower burnout and better well-being in demanding occupations (Schaufeli et al., 2006). In policing, these mechanisms are particularly relevant given the high emotional demands of the job.

Furthermore, emotional regulation has been shown to contribute to the development of resilience, strengthening the ability to process negative experiences appropriately (Arnetz et al., 2009). This suggest that emotional competencies not only benefit police intervention, but also have a cumulative effect on psychological adaptation throughout one’s professional career.

### 3.4 Sense of purpose and professional identity

The meaning that officers attribute to their work is another factor that the literature describes as particularly relevant to psychological well-being. Numerous studies show that police officers who perceive their work as valuable, aligned with their own values or with a sense of public mission, show lower levels of burnout and greater intrinsic motivation (Bakker et al., 2005; Kuo, 2015).

Professional identity, especially in organisations with a strong organisational culture, can act as a protective resource against frustration or fatigue. When agents recognise the positive impact of their work or receive external validation, they tend to experience greater job satisfaction, even in complex environments.

However, some studies warn that when there is a discrepancy between the aspirational purpose of police work and the actual conditions of performance—for example, bureaucratic burden, lack of recognition, or tensions with the public—feelings of cynicism, detachment, or demotivation may arise (Skogan and Meares, 2004).

### 3.5 Organisational climate, leadership and organisational culture

The final thematic area concerns the working environment and leadership, two elements that have a decisive influence on how officers perceive and manage their professional experience. The relationship between leadership and police well-being has been the subject of increasing attention over the last decade. A significant proportion of the studies analysed indicate that more authoritarian, rigid or distant leadership styles are associated with higher levels of stress and internal conflict, while more participatory and transformational approaches tend to promote a healthier environment (Manzoni and Eisner, 2006; Aymerich et al., 2021).

The organisational climate also includes factors such as organisational justice, internal communication, clarity of protocols, and professional recognition. A positive climate promotes cohesion and reduces the perception of overload, while a negative climate can intensify feelings of isolation and exacerbate the impact of operational demands (Tucker, 2015).

The body of reviewed work suggests that, although individual competencies are important, organisational factors have an equal or greater weight in preventing psychological distress. This reinforces the idea that interventions should target both agents and their managers and institutional structures. This approach is in line with the research carried out by Leiter and Maslach (2004, 2016), who have pointed out that burnout and psychosocial risks should be analysed as organisational phenomena rather than individual failures.

## 4 Discussion

The purpose of this narrative review was to synthesise existing evidence on protective factors for police mental health and to integrate these findings into an applied conceptual framework to guide prevention-oriented strategies in policing contexts. The discussion that follows interprets the reviewed literature in light of this objective, highlighting how individual, organisational and cultural factors interact to promote psychological well-being.

The results of this narrative review show that mental health in the police force does not depend solely on individual factors, but rather emerges from a complex interaction between personal, organisational and cultural elements. Although resilience, emotional regulation, and sense of purpose provide important resources at the individual level, the reviewed literature suggests that the institutional environment and the quality of leadership have a decisive influence on the psychological experience of officers (Aymerich et al., 2021). This conclusion coincides with recent trends in occupational psychology, which emphasise the need for systemic and organisational models to understand well-being in high-risk professions (Leiter and Maslach, 2016).

A relevant aspect is that many of the studies reviewed describe a cumulative pattern of stress. Officers are not usually affected by a single traumatic event, but rather by the sum of small daily stresses: long shifts, constant conflict management, repeated exposure to human suffering, and the pressure inherent in making quick decisions. This accumulation contributes to high levels of emotional fatigue and professional burnout, especially when adequate support structures are lacking (Violanti et al., 2017; Garbarino et al., 2013). Hence, prevention programmes must consider not only immediate coping after critical incidents, but also the impact of everyday working conditions.

Similar patterns have been reported in other public service sectors. Recent evidence from primary care professionals indicates that exposure to workplace aggression is strongly associated with post-traumatic stress symptomatology and burnout (Gascon-Santos et al., 2024), suggesting that aggression-related stress represents a shared challenge across frontline professions.

Resilience appears to be a promising line of intervention; however, evidence from both cross-sectional and longitudinal studies reviewed suggests that individual resilience alone is insufficient to prevent chronic stress when organisational stressors persist. Studies examining perceived organisational support, leadership style and work climate consistently report that resilient officers may still experience emotional exhaustion and burnout in contexts characterised by high demands, low support or insensitive leadership (e.g., Shane, 2010; Papazoglou and Andersen, 2014; Violanti et al., 2017).

In this regard, organisational support is consolidated as one of the most robust predictors of police well-being. The perception of being supported by the institution—especially by direct superiors— cushions the impact of operational stress and significantly reduces the risk of burnout (Ménard and Arter, 2014). The role of middle managers is particularly relevant, as they act as a bridge between the strategic requirements of the institution and the operational reality of the officer. Their ability to recognise effort, encourage communication and create safe environments largely determines how the professional experience is lived.

Another important finding is the growing relevance of emotional literacy in police forces. Although historically this aspect has been neglected in police training, research agrees that emotional regulation improves the ability to de-escalate situations, reduces impulsivity, and protects against emotional exhaustion (Schaufeli et al., 2006). Training programmes that incorporate these skills have demonstrated positive effects on the ability to manage intense emotions and reduce conflicts during operational interventions (Arnetz et al., 2009). These results suggest that emotional literacy should not be considered a complement, but rather a central component of ongoing training.

A sense of purpose emerges as another key element. Many officers experience a strong professional identity linked to public service, justice, and the protection of citizens. When this identity is reinforced through institutional recognition, work becomes more meaningful and the impact of stress decreases (Bakker et al., 2005; Kuo, 2015). However, several authors warn that the gap between the ideal of police work and operational reality can lead to feelings of frustration or cynicism, especially in contexts of high bureaucracy or lack of social support (Skogan and Meares, 2004). This makes it necessary for institutions to foster a culture in which professional purpose is not diluted by administrative demands or external tensions.

Finally, the studies reviewed agree that the psychosocial climate and leadership significantly influence staff mental health. The presence of transformational leadership—based on communication, empathy, and shared vision—is associated with greater cohesion, less stress, and a better perception of work (Aymerich et al., 2021). In contrast, authoritarian or overly hierarchical styles can intensify emotional distress and feelings of lack of control. At this point, it is clear how important it is to train managers not only in tactical matters, but also in emotional management skills and organisational well-being.

While the evidence converges on the protective value of resilience, organisational support, emotional literacy and healthy leadership, translating these principles into practice is not straightforward in many police organisations. Research and institutional reports have long highlighted police mental health as an enduring challenge, yet the development of internal support networks and prevention-oriented policies has often been slow. This delay is partly explained by entrenched organisational cultures characterised by rigid hierarchies, command-and-control management styles, and limited psychological safety for officers to disclose distress. In parallel, stigma—both perceived and internalised—continues to act as a major barrier to help-seeking, reducing the uptake of available services even when they exist. For these reasons, the IPMHM should be understood not only as a technically sound framework, but also as a change-management proposal that requires deliberate implementation strategies. In practice, successful adoption is more likely when interventions are introduced in phased formats (e.g., pilot units), accompanied by visible senior leadership endorsement, confidentiality safeguards, and peer-support structures that normalise early assistance. Embedding these components into training and supervisory routines may increase feasibility and reduce resistance, particularly in contexts where organisational inertia and stigma remain significant.

Taken together, these findings suggest that promoting mental health in police forces requires a comprehensive strategy that combines individual strengthening with group interventions and structural improvements. The convergence of scientific evidence around these five areas provides the basis for the design of the Integrated Police Mental Health Model (IPMHM), which aims to offer a practical framework that can be applied to different police realities.

This review has several limitations that should be acknowledged. First, as a narrative review, it does not aim to provide an exhaustive or systematically weighted synthesis of all available studies, nor does it quantify effect sizes or causal relationships. Second, the included literature is predominantly drawn from European and North American policing contexts, which may limit the transferability of some findings to other institutional or cultural settings. Finally, although the review integrates evidence across multiple thematic domains, it does not evaluate the effectiveness of specific interventions, highlighting the need for future empirical studies to test and refine the proposed model in applied settings.

## 5 Proposal for an Integrated Police Mental Health Model (IPMHM)

The results of the review show that police mental health is determined by a network of individual, group and organisational factors. Based on this evidence, the Integrated Model of Police Mental Health (IPMHM) is proposed, a conceptual framework aimed at facilitating the planning of preventive strategies, training programmes and institutional actions designed to strengthen the psychological well-being of police personnel. The model is organised into five interdependent pillars (Table 3): resilience, organisational support, emotional literacy, sense of purpose, and work climate. Each of these can be developed through specific interventions that can be adapted to different police forces.

**Table 3:** Pillars of the Integrated Police Mental Health Model (IPMHM) and main components.

Pillar of the IPMHM	General description	Key components	Practical applications in police forces
1. Dynamic resilience	Ability to adapt and recover in critical situations and under operational stress, both individually and as part of a team.	Active coping Cognitive restructuring Unity cohesion Psychological recovery techniques	Training in high- pressure simulations Psychological strengthening programmes Structured debriefings after critical incidents.
2. Social and organisational support	Support networks among colleagues, supervisors, and institutional structures that cushion the impact of stress.	Peer support Empathetic supervision Post-incident protocols Organizational recognition	Teams trained in emotional support Open-door policy among management Access to psychological services Work-life balance policies.
3. Emotional literacy and affective regulation	Skills to identify, understand and regulate one’s own and others’ emotions during interventions and in everyday working life.	Emotional self- awareness Physiological regulation Conflict management Emotional communication.	Training in emotional intelligence Operational mindfulness Self-control techniques Communication workshops
4. Sense of purpose and professional identity.	Agent connection with the social value of their work, the institutional mission, and service identity.	Clear professional values Meaning of work Social recognition Coherent institutional narratives	Recognition programmes Professional reflection spaces Feedback on the social impact of work Mentoring
5. Psychosocial climate, leadership, and organisational culture	Work environment and leadership style influencing the emotional and operational experience of the agent.	Organisational justice Internal communication Transformational leadership Clear procedures	Emotional training for managers Assessment of the working environment Support protocols Staff participation systems

Source: prepared internally.

### 5.1 Pillar 1: dynamic resilience

Resilience is the structural backbone of the model, but it is not understood as a static attribute, but rather as a trainable capacity that is strengthened through experience, training, and context. In the police field, resilience involves facing risky situations without losing the ability to analyse, remaining calm in uncertain scenarios, and regaining balance after emotionally intense interventions.

The most effective resilience programmes combine cognitive reinterpretation exercises, problem- solving training, simulations of decision-making under pressure, and psychological recovery techniques after critical incidents (Arnetz et al., 2009; Papazoglou and Andersen, 2014). In addition, collective resilience—derived from group cohesion and trust within the unit—acts as an additional buffer against operational stress. For this reason, the IPMHM proposes dual interventions, both individual and group-based.

### 5.2 Pillar 2: social support and organisational support

The second pillar of the model focuses on the role of interpersonal and organisational support, which evidence identifies as one of the strongest predictors of police well-being. This pillar encompasses three levels of intervention.

1. Peer support: police officers trained to provide emotional support to colleagues who are experiencing intense stress or who have just been through a critical incident.
2. Support from supervisors and middle managers: leadership styles that prioritise communication, active listening and emotional availability.
3. Institutional support: clear work-life balance policies, access to psychological services, professional recognition and protocols for action after critical incidents.

Numerous studies have shown that agents who perceive support at these three levels have a lower prevalence of burnout, greater job satisfaction, and greater organisational commitment (Shane, 2010; Ménard and Arter, 2014).

### 5.3 Pillar 3: emotional literacy and affective regulation

The IPMHM incorporates a pillar focused on emotional competencies, an area that, although historically neglected in police training, has been shown to play a decisive role in stress management and decision-making. Emotional literacy includes:

- Identify and understand one’s own emotions and those of others,
- Regulate the emotional impact of critical situations,
- Maintain self-control in interactions with citizens,
- Prevent impulsive reactions.

Training in this area usually combines mindfulness practices adapted to the police environment, physiological regulation exercises, analysis of real cases, and training in emotional communication. Evidence shows that police officers who develop these skills experience less emotional exhaustion and show a greater ability to de-escalate conflicts (Schaufeli et al., 2006).

### 5.4 Pillar 4: sense of purpose and professional identity

The fourth pillar relates to the more vocational dimension of police work. A sense of purpose acts as a psychological anchor that helps officers interpret their work as a valuable contribution to the community. When this purpose is aligned with everyday practice and supported by the organisation, it tends to lessen the impact of stress and increase intrinsic motivation.

The IPMHM proposes three lines of intervention:

1. Reinforcing the social impact of police work through internal campaigns, testimonials, and genuine recognition.
2. Positive organisational narratives that link police work with values of service, protection, and justice.
3. Feedback processes where officers can express their perceptions of their role and adjust work expectations.
4. Various studies suggest that a well-articulated sense of purpose helps reduce cynicism, detachment and frustration associated with the emotional burden of work (Bakker et al., 2005; Kuo, 2015).

### 5.5 Pillar 5: psychosocial climate, leadership and organisational culture

The fifth pillar is dedicated to the institutional environment, which has a decisive influence on how agents experience their work. This includes elements such as leadership style, organisational justice, internal communication and clarity of procedures.

A significant portion of the studies reviewed indicate that managers with a transformational approach—based on empathy, vision, and collaboration—create units with less stress, greater cohesion, and lower turnover (Aymerich et al., 2021). Conversely, authoritarian styles tend to intensify discomfort and hinder emotional expression.

The IPMHM proposes interventions that include:

- Emotional training for managers
- Regular assessment of the working environment,
- Recognition systems not solely linked to productivity,
- Support protocols following critical events,
- Consultation spaces where agents can contribute suggestions for improvement.

A healthy organisational climate not only protects mental health, but also improves operational efficiency and strengthens trust between officers and citizens.

## 6 Conclusion

The review shows that the mental health of police personnel cannot be understood solely from the perspective of risk or the emotional impact of critical situations. Although these elements are relevant, the psychological well-being of officers is built—and sustained—through a balance between individual resources, social support, organisational structure, and professional culture. A significant portion of the studies analysed suggest that when these factors are aligned, police officers cope more effectively with the demands of their work and experience higher levels of satisfaction, emotional stability, and a sense of belonging.

The findings of this review reinforce the need to move beyond individualised approaches to police mental health and towards integrated, prevention-oriented strategies. Leadership, organisational support and emotional competencies emerge not as peripheral elements, but as central determinants of well-being and sustainable performance in policing contexts.

From a practical perspective, the IPMHM offers a structured framework to guide training, leadership development and organisational policies aimed at strengthening psychological well-being. Its value lies in integrating evidence-based protective factors into a coherent model that can be adapted to different police forces and institutional realities.

Future research should focus on empirically evaluating the implementation of this model, examining its feasibility, acceptability and impact over time. Such efforts would contribute not only to improving occupational health among police officers, but also to enhancing the quality and sustainability of policing services.

Finally, the feasibility of implementing integrated wellbeing frameworks depends on organisational readiness; therefore, cultural change, leadership commitment and stigma reduction should be considered core enabling conditions for the IPMHM.
